# YAP integrates the regulatory Snail/HNF4α circuitry controlling epithelial/hepatocyte differentiation

**DOI:** 10.1038/s41419-019-2000-8

**Published:** 2019-10-10

**Authors:** Valeria Noce, Cecilia Battistelli, Angela Maria Cozzolino, Veronica Consalvi, Carla Cicchini, Raffaele Strippoli, Marco Tripodi, Alessandra Marchetti, Laura Amicone

**Affiliations:** 1 Istituto Pasteur-Fondazione Cenci Bolognetti, Department of Molecular Medicine, Sapienza University of Rome, Rome, Italy; 2 National Institute for Infectious Diseases L. Spallanzani, IRCCS, Rome, Italy

**Keywords:** Cell biology, Molecular biology

## Abstract

Yes-associated protein (YAP) is a transcriptional co-factor involved in many cell processes, including development, proliferation, stemness, differentiation, and tumorigenesis. It has been described as a sensor of mechanical and biochemical stimuli that enables cells to integrate environmental signals. Although in the liver the correlation between extracellular matrix elasticity (greatly increased in the most of chronic hepatic diseases), differentiation/functional state of parenchymal cells and subcellular localization/activation of YAP has been previously reported, its role as regulator of the hepatocyte differentiation remains to be clarified. The aim of this study was to evaluate the role of YAP in the regulation of epithelial/hepatocyte differentiation and to clarify how a transducer of general stimuli can integrate tissue-specific molecular mechanisms determining specific cell outcomes. By means of YAP silencing and overexpression we demonstrated that YAP has a functional role in the repression of epithelial/hepatocyte differentiation by inversely modulating the expression of Snail (master regulator of the epithelial-to-mesenchymal transition and liver stemness) and HNF4α (master regulator of hepatocyte differentiation) at transcriptional level, through the direct occupancy of their promoters. Furthermore, we found that Snail, in turn, is able to positively control YAP expression influencing protein level and subcellular localization and that HNF4α stably represses YAP transcription in differentiated hepatocytes both in cell culture and in adult liver. Overall, our data indicate YAP as a new member of the HNF4/Snail epistatic molecular circuitry previously demonstrated to control liver cell state. In this model, the dynamic balance between three main transcriptional regulators, that are able to control reciprocally their expression/activity, is responsible for the induction/maintenance of different liver cell differentiation states and its modulation could be the aim of therapeutic protocols for several chronic liver diseases.

## Introduction

YAP (Yes-associated protein, Yki ortholog) is a transcriptional co-factor able to regulate a large number of genes involved in several cell processes, including proliferation, differentiation, organ size control and maintenance of stemness traits in embryonic and cancer stem cells^1^. YAP is the downstream effector of the Hippo pathway, an oncosuppressor signaling known to be altered in various human tumors^2^. Hippo activity depends on several extra- and intracellular stimuli and involves the function of MSTs and LATS1/2 kinases that target YAP thus causing its cytoplasmic sequestration and proteasome degradation^3–7^. Inversely, when Hippo pathway is inactivated, the un-phosphorylated form of YAP translocates into the nucleus where, in association with transcription factors mainly belonging to the TEAD/TEF family, activates several genes, some of which with functions yet unclear^8^.

Regarding cell differentiation, YAP nuclear localization and its transcriptional activity have been related either to proliferation and maintenance of stem/progenitor cells or to activation of specific differentiation programs. In mice, YAP overexpression provokes dedifferentiation of intestinal cells together with an expansion of stem cell compartment^9^. Similarly, YAP overexpression in skin alters cell stratification impairing terminal differentiation of keratinocytes^10^. On the other hand, an active nuclear YAP correlates with the differentiation of mesenchymal stem cells toward osteoblasts^11,12^ as well as with the astrocyte differentiation from neural stem cells^13^. Furthermore, it has been reported that an increase of YAP level induces epithelial-to-mesenchymal transition (EMT) mainly involved in epithelial cancer progression^14^.

In the adult liver, the activity of Hippo pathway controls liver cell fate, as suggested by studies in murine models, where its complete inactivation induces hepatocyte dedifferentiation^15^. Moreover, it has been recently demonstrated that, during mouse liver development, Hippo signaling controls the shift of gene expression from hepatoblasts to hepatocytes by influencing the redistribution of master transcriptional factors on a wide range of promoters and enhancers^16^.

On the other hand, the exogenous expression of YAP induces hepatocyte proliferation and organ hyperplasia together with subversion of the normal metabolic zonation, acquisition of cholangiocyte markers in hepatocytes, and expansion of progenitor cell compartment^17^.

One of the main stimuli inducing Hippo inactivation and YAP nuclear translocation is the mechanical stress transmitted from extracellular environment to cytoskeleton, by cell stretching or extracellular matrix (ECM) increased rigidity^18^. In the liver, the normal organ stiffness dramatically increases during fibrosis, a pathological condition resulting from chronic injury, including viral and toxic hurts^19^. As suggested by studies of hepatocyte functions in cells cultured on substrates of different elasticity, the increase of ECM rigidity in vivo, other than to subvert the liver circulatory dynamics, can directly affect hepatocyte differentiation and function. We have recently shown that (i) manipulation of substrate stiffness influences the differentiation of liver progenitor cells as well as the functionality of hepatocytes and (ii) YAP subcellular localization/activity correlates with different differentiation states and functions of liver cells^20^. However, while YAP has been well described as an intracellular mechanical rheostat, little work has been done so far to understand how it integrates general extracellular cues into liver-specific molecular mechanisms and cell outcome.

We previously demonstrated that two master factors, Snail and HNF4α, play a pivotal role in the metastability of liver stem/precursor cells, in the maintenance of epithelial/hepatocyte phenotype and in the dynamic events of EMT and mesenchymal-to-epithelial transition (MET)^21–23^. Snail is a well-characterized transcriptional inhibitor that acts as a master regulator of the mesenchymal program in EMT and in liver stemness^22,24^. Moreover, its expression in tumor cells correlates with a more aggressive and metastatic phenotype^25–27^. HNF4α is a transcriptional factor able to orchestrate the expression of several epithelial markers in hepatocytes^28^ as well as to confer to fibroblasts an epithelial-like morphology^29^ and to re-establish a differentiated phenotype in invasive hepatocellular carcinoma cells, both in vivo and in vitro^30^. This latter ability granted the role of MET master gene to HNF4α. We previously showed that HNF4α and Snail are part of a liver-specific mini-circuitry of reciprocal inhibition whose balance is responsible for different cell outcomes (i.e., differentiation vs stemness; MET vs EMT; tumor suppression vs tumor progression)^21–23,31^. Notably, we also described a correlation among HNF4α, Snail and YAP expression levels during dynamic differentiation of liver stem/progenitor cell lines toward hepatocytes, obtained in traditional cell culture conditions as well as culturing cells on low stiffness hydrogel^20,22^.

Data presented here show a direct role of YAP in the repression of liver cell differentiation through the opposite regulation of Snail and HNF4α. In particular, gene expression analysis performed in condition of YAP silencing and YAP overexpression, together with ChIP assays exploring the dynamic recruitment of YAP on Snail and HNF4α gene regulatory regions, showed a direct involvement of YAP in Snail gene upregulation and HNF4α gene repression. Interestingly, we also observed that Snail and HNF4α, in turn, are able to influence YAP expression/activity in opposite manner. In particular, HNF4α was shown to be stably recruited on YAP promoter and to repress its expression both in hepatocyte cell lines and in adult livers.

In conclusion, our data point to YAP as a new leading player of liver cell differentiation process. It integrates an epistatic molecular circuitry of liver-specific transcriptional regulation, in which the balance between three different master regulators controls liver cell state.

## Results

### YAP nuclear localization and activity inversely correlate with hepatocyte differentiation in liver cell lines

Our recently published data showed the pivotal role played by different ECM stiffness in the maintenance of liver cell stemness and in the induction/maintenance of hepatocyte differentiation as well as the correlation between ECM rigidity, specific cell outcome and YAP localization/activity^20^.

Moreover, YAP activity has been demonstrated to be influenced by several other stimuli known to impact on hepatocyte differentiation, such as hormonal signals acting through G-protein-coupled and tyrosine kinase receptors^32,33^, and to play a pivotal role in EMT^14^, a process responsible for hepatocyte dedifferentiation in vivo and in vitro.

Starting from these observations, we investigated a possible direct involvement of YAP in the induction and maintenance of epithelial/hepatocyte differentiation of liver cells and the molecular mechanisms involved.

**Fig. 1:**
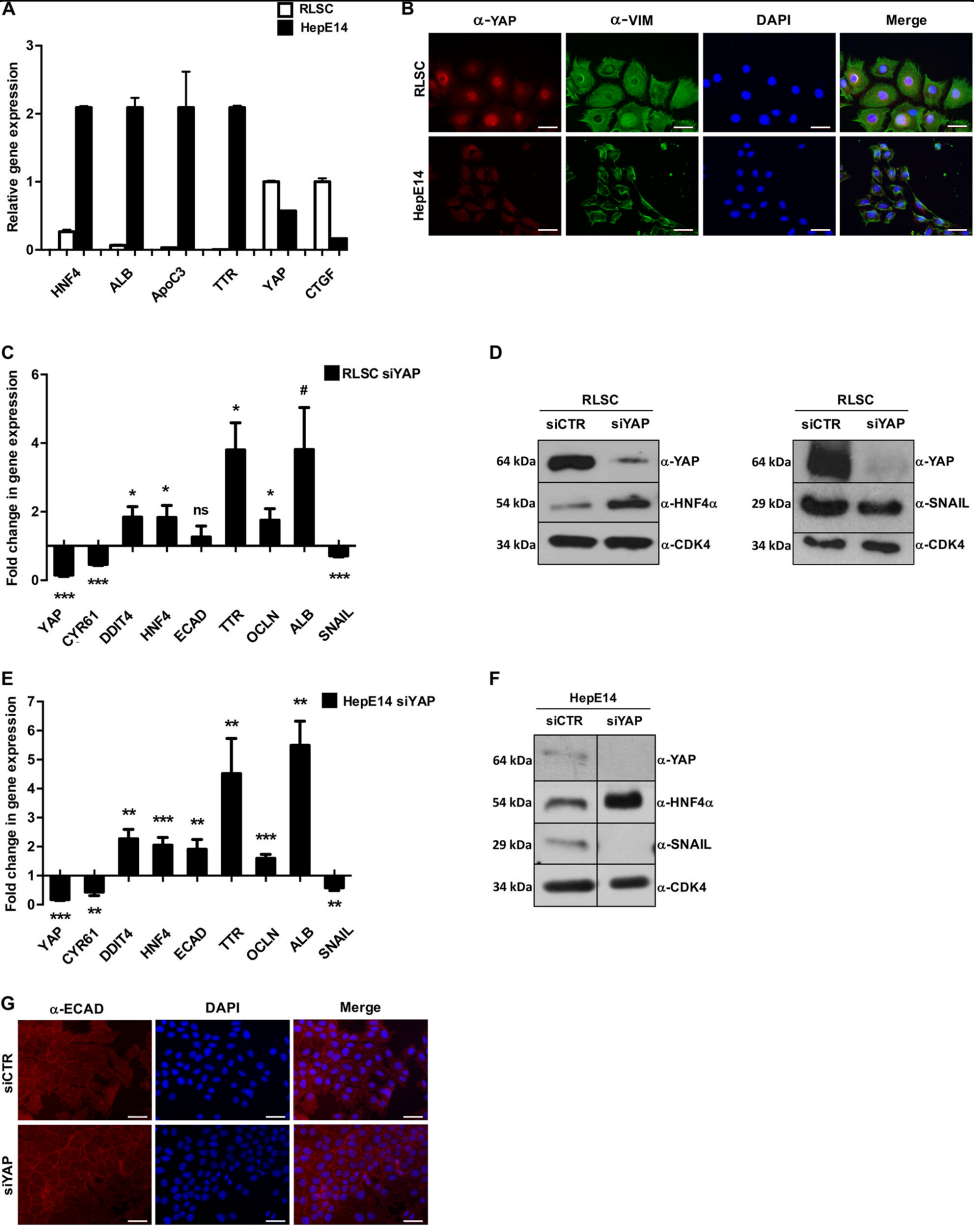
YAP silencing positively impacts on epithelial/hepatocyte differentiation. **a** RT-qPCR analysis for the indicated genes on RLSC and HepE14 cell lines. **b** Immunofluorescence analysis for YAP (red) and the mesenchymal marker Vimentin (VIM, green) in RLSC and HepE14 cell lines. Nuclei were stained with DAPI (blue). Images are representative of three independent cell cultures. Scale bar: 50 µm. **c** RT-qPCR analysis for the indicated genes in YAP-silenced RLSCs (RLSC siYAP), compared with GFP-silenced control cells (RLSC siCTR). The values are calculated by the 2^(−ΔCt)^ method, expressed as fold of expression versus the control (arbitrary value = 1) and shown as means ± S.E.M. of five independent experiments. Statistically significant differences are reported (*p < 0.05, ***p < 0.001; ^#^*p* = 0.052; ns = not significant). **d** Western blot analysis for YAP, HNF4α and Snail in RLSC siYAP and RLSCs siCTR. CDK4 was used as loading control. **e** RT-qPCR analysis for the indicated genes in YAP-silenced hepatocytes (HepE14 siYAP), compared with control GFP-silenced cells. The values, calculated as in (**c**), are shown as means ± S.E.M. of at least five independent experiments. Statistically significant differences are reported (**p < 0.01, ***p < 0.001). **f** Western blot analysis for YAP, HNF4α and Snail in HepE14 siYAP and HepE14 siCTR. CDK4 was used as a loading control. **g** Immunofluorescence analysis of E-cadherin (ECAD, red) in HepE14 siYAP, compared with HepE14 siCTR. Nuclei were stained with DAPI (blue). Images are representative of three independent experiments. Scale bar: 50 µm

In order to verify the correlation between YAP subcellular localization/activity and cell differentiation state, independently from the substrate stiffness, we analyzed two liver cell lines, largely characterized in our laboratory, as models of liver stem/progenitor cells (RLSCs, from resident liver stem cells) and of functional hepatocytes (HepE14)^34–36^. The analysis of hepatocyte-specific transcriptional profile (including HNF4α, albumin, apolipoprotein-C3, and transthyretin genes), in RLSC and HepE14 cells showed a strict correlation between hepatocyte differentiation (Fig. 1a) and YAP state (Fig. 1a, b). In fact, YAP is expressed, properly located in the nucleus and active on its major positive target gene, connective tissue growth factor (CTGF)^37^, in undifferentiated cells. Conversely, only a low level of transcripts and scarce protein, mainly localized into the cytoplasm, were detectable in hepatocytes (Fig. 1a, b).

This result, besides confirming the inverse correlation between YAP activity and hepatocyte differentiation, that we previously observed by culturing cells on different ECM stiffness^20^, also indicated that RLSC and HepE14 cell lines are suitable cell models to investigate the role played by YAP in the process of liver cell differentiation.

### YAP has a functional role in the repression of epithelial/hepatocyte differentiation by regulating the expression of EMT and MET master genes in opposite manner

Being YAP subcellular localization and activity related to different liver cell states, we formally proved its role in hepatocyte differentiation, by means of experiments of YAP silencing and overexpression performed in mesenchymal like/undifferentiated RLSC and epithelial/differentiated HepE14 cell lines, respectively.

In YAP-silenced RLSCs, the expression of the epithelial/hepatocyte master gene HNF4α, the liver function genes transthyretin (TTR) and albumin (ALB), and the epithelial gene occludin (OCLN) resulted upregulated, while the mesenchymal marker and EMT master gene Snail resulted significantly decreased (Fig. 1c). The inverse modulation of HNF4α and Snail, the MET and EMT master genes, respectively, was confirmed at protein level (Fig. 1d). As expected, YAP silencing induced the upregulation of DNA-damage-inducible transcript 4 (DDIT4), a negative YAP-target gene, and the downregulation of cysteine rich protein 61 (Cyr61), a positive YAP-target gene, both involved in opposite manner in the regulation of stemness/cancer properties in epithelial cells^38,39^ (Fig. 1c). However, YAP silencing resulted insufficient to induce in RLSCs a fully differentiated phenotype, as demonstrated by the slight increase of the cell–cell adhesion molecule E-cadherin and the maintenance of an undifferentiated morphology (data not shown). Instead, the experimental neutralization of the residual YAP activity in HepE14 cells enhanced the epithelial/hepatocyte differentiation, as assessed by the transcriptional profile and by the massive increase of HNF4α protein level (Fig. 1e, f). Moreover, a significant induction of E-cadherin expression and its localization to the plasma-membrane were observed, ultimately causing a strengthening of the epithelial phenotype (Fig. 1g).

Overall, this data indicates that YAP downregulation represents a crucial event in the triggering of the differentiation process in progenitor cells and in the accomplishment of a full epithelial/hepatocyte program in cultured hepatocytes.

**Fig. 2:**
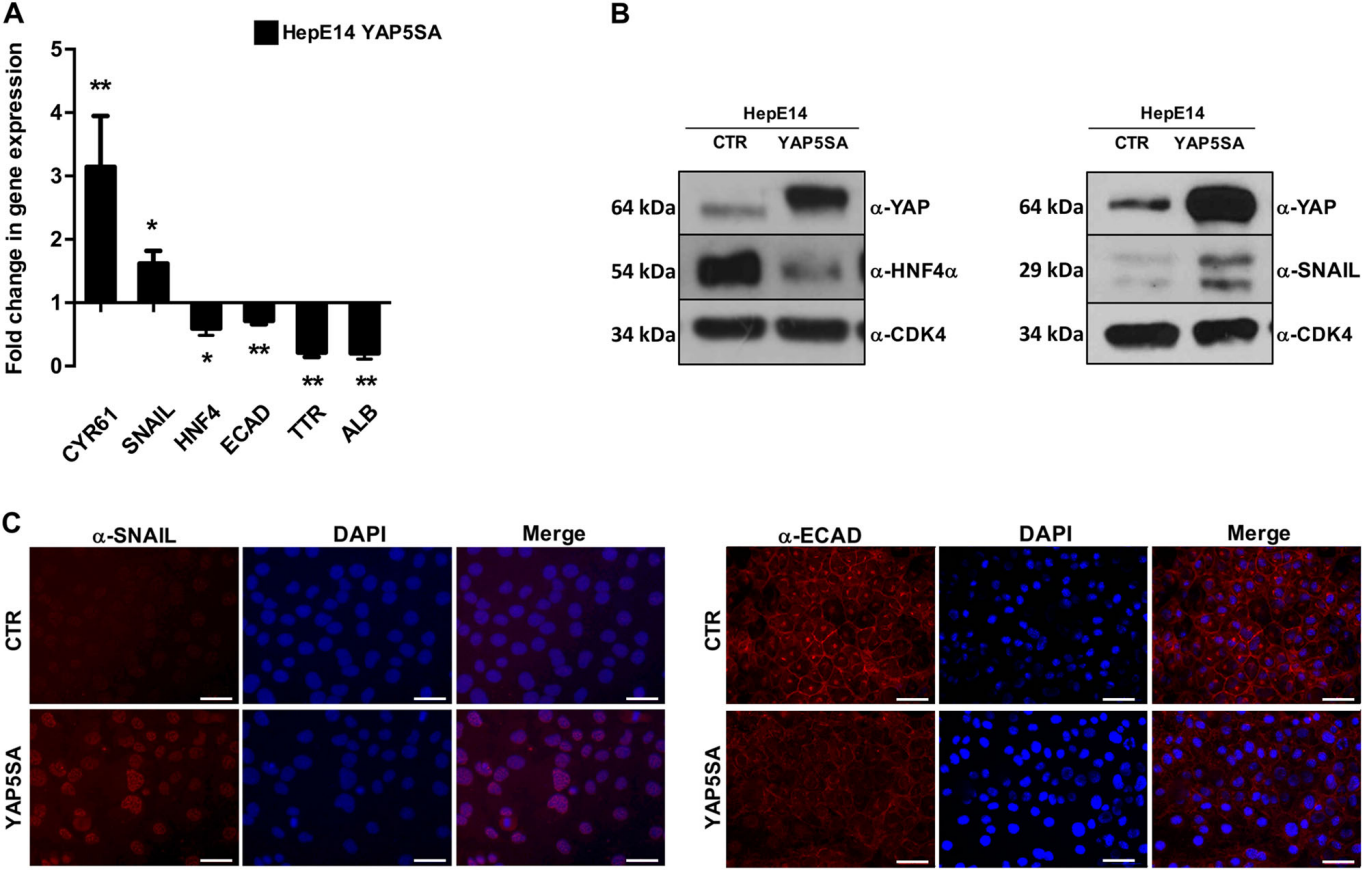
YAP overexpression hampered HepE14 differentiation state. **a** RT-qPCR analysis for the indicated markers in HepE14 transiently transfected with pQCXIH-Myc-YAP-5SA (HepE14 YAP5SA), compared with control cells transfected with empty vector (HepE14 CTR). The values are calculated by the 2^(−ΔCt)^ method, expressed as fold of expression versus the control (arbitrary value = 1) and shown as means ± S.E.M. of at least three independent experiments. Statistically significant differences are reported (*p < 0.05; **p < 0.01). **b** Western blotting analysis for YAP, HNF4α and Snail in HepE14 YAP5SA and HepE14 CTR. CDK4 was used as a loading control. **c** Immunofluorescence analysis of Snail and E-cadherin in HepE14 YAP5SA, is compared with HepE14 CTR. Nuclei were stained with DAPI (blue). Images are representative of three independent experiments. Scale bar: 50 µm

Coherently with the results obtained in experiments of silencing, the overexpression in HepE14 cells of a constitutively active mutant of YAP (i.e., resistant to LATS1/2-dependent inhibitory phosphorylations), named YAP5SA^7^, produced a significant transcriptional downregulation of HNF4α, E-cadherin, TTR, and albumin genes (Fig. 2a), together with a massive reduction of HNF4α protein level (Fig. 2b). In addition, a significant upregulation of Snail transcripts (Fig. 2a), and protein (Fig. 2b) can be observed. Snail protein, moreover, appeared localized in the nucleus (Fig. 2c, left panels), where most likely was responsible for the transcriptional downregulation of E-cadherin, its well-known target gene. The E-cadherin transcriptional inhibition, together with the delocalization from plasma-membrane of the residual protein, correlated with an impairment of the epithelial cell phenotype (Fig. 2c, right panels).

Notably, to increase the significance of these results and to generalize the observations, experiments of YAP overexpression have been performed in two additional functional hepatocyte cell lines, in which a similar modulation of gene expression has been observed (Supplementary Fig. S1).

Altogether, these findings demonstrated a key role of YAP in the negative control of epithelial/hepatocyte differentiation and suggested the transcriptional control of the master genes HNF4α and Snail, as one of the major regulative mechanisms involved.

### YAP directly controls Snail and HNF4α transcription

**Fig. 3:**
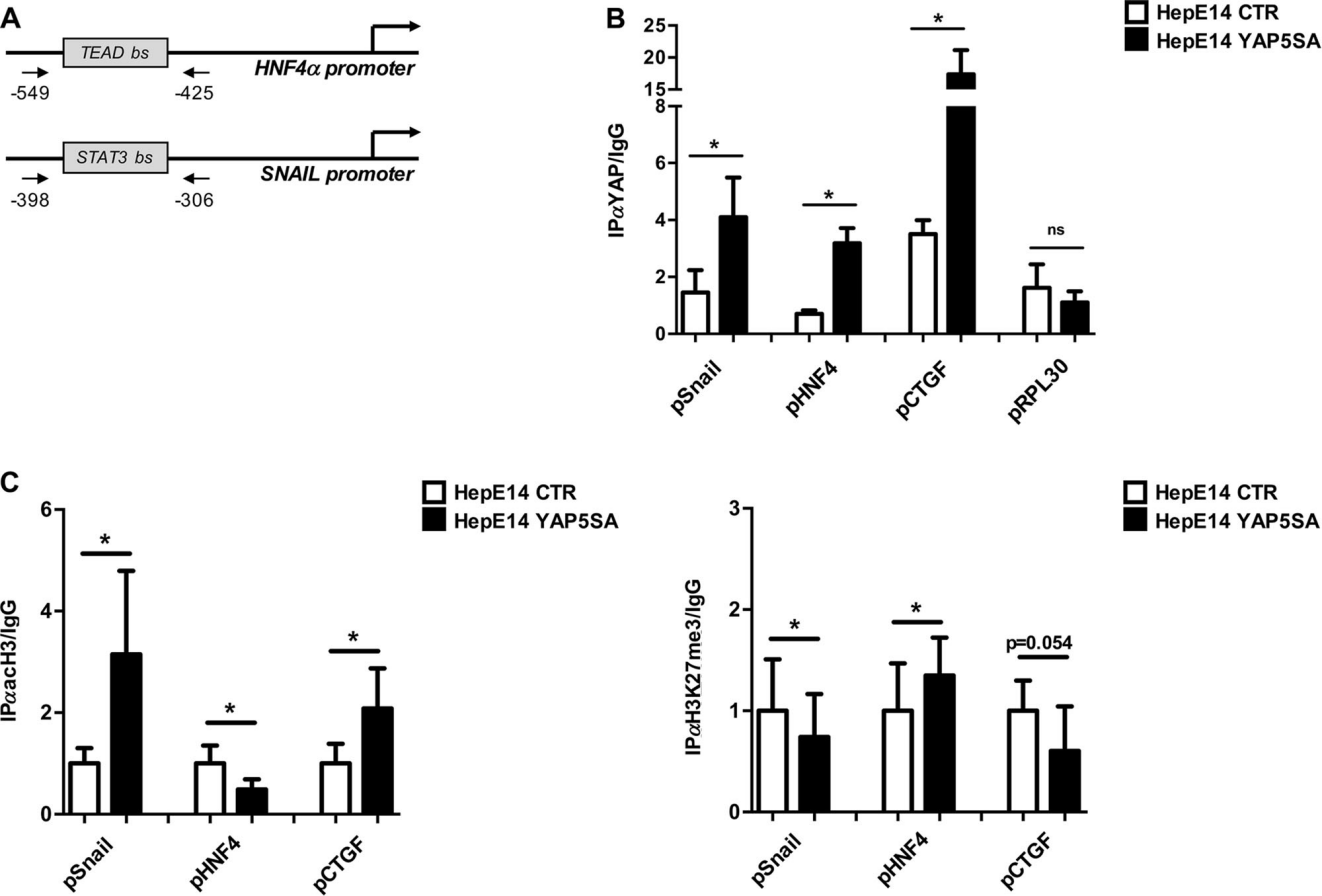
YAP directly regulates Snail and HNF4α transcription. **a** Schematic representation of murine HNF4α P1 and Snail promoters. The amplified regions are indicated by arrows. **b** qPCR analysis of ChIP assays with α-YAP antibody, and as control, with normal rabbit IgG on chromatin from HepE14 CTR or HepE14 YAP5SA. TEAD consensus binding site on the murine CTGF promoter was used as positive control. RPL30 promoter is used as negative control. Values derived from at least three independent experiments are calculated as IP/IgG and reported as means ± S.E.M. Statistically significant differences are reported (*p < 0.05; ns = not significant). **c** qPCR analysis of ChIP assays with α- acetyl H3 antibody (acH3), α-H3K27me3 antibody, and as control, with normal rabbit IgG on the same chromatin shown in (**b**). Values derived from at least three independent experiments are calculated as IP/IgG and reported as means ± S.E.M. respect to the control sample (arbitrary value = 1). The TEAD consensus site on the promoter of the YAP positive target gene CTGF was used as control of YAP-dependent chromatin modifications. Statistically significant differences are reported (*p < 0.05)

YAP is recruited on target genes by transcription factors, mainly those belonging to TEAD family^40^. Therefore, to investigate whether, in liver cells, YAP regulates Snail and HNF4α expression at transcriptional level, we accomplished a preliminary analysis of gene promoters by Genomatix MatInspector, looking for putative TEAD binding sites. Concerning HNF4α, previous studies demonstrated that YAP can be recruited on a TEAD binding site located in the first intron of the HNF4α gene body during liver development^41^; however, in RLSCs, this region was unable to recruit YAP (data not shown). Thus, by MatInspector analysis we looked for other putative TEAD binding sites on HNF4α promoter. A non-canonical TEAD consensus site (5′-AAGCATGT-3′), located at −518 from the transcription start site (TSS), was found (Fig. 3a, upper panel).

Regarding Snail promoter, within 1500 bp upstream the TSS we did not find any TEAD consensus motif, while we identified a STAT3 binding site located at −350 from TSS (Fig. 3a, lower panel). Since literature data demonstrated the ability of YAP to physically interact and to cooperate with STAT3 in the transcriptional activation in endothelial cells^42,43^, we decided to analyze the YAP occupancy of this region.

As shown in Fig. 3b, a chromatin immunoprecipitation (ChIP) assay in HepE14 cells overexpressing YAP5SA protein, unveiled a significant recruitment of YAP to the chromatin region surrounding STAT3 binding site of Snail promoter and to the non-canonical TEAD consensus of HNF4α promoter. We used CTGF and RPL30 promoters as positive and negative controls of YAP recruitment, respectively. Moreover, and in accordance with the transcriptional upregulation of Snail and downregulation of HNF4α by YAP shown above, we observed coherent variations of the corresponding chromatin modifications. In particular, the acetylation of the histone H3 (H3Ac), an activating chromatin modification, around STAT3 consensus on Snail promoter was significantly higher in HepE14 cells overexpressing YAP compared with control cells. On the contrary, the acetylation of HNF4α promoter resulted significantly lower (Fig. 3c, left panel). Accordingly, the main repressive chromatin modification, the trimethylation of lysine 27 of histone H3 (H3K27me3) on HNF4α promoter was significantly increased by the YAP binding, while the same modification on Snail promoter appeared reduced (Fig. 3c, right panel). CTGF promoter was used as control of YAP-dependent chromatin modifications.

Overall, the finding of the recruitment of YAP on Snail and HNF4α promoters, and the observed coherent chromatin modifications, confirmed the involvement of YAP in the transcriptional regulation of HNF4α and unveiled Snail as a new transcriptional target of YAP.

We also identified a new binding site of TEAD/YAP on HNF4α promoter, and more importantly, a site of YAP recruitment on Snail promoter, including a STAT3 consensus. To enforce the significance of this last observation, we investigated on the possible crosstalk between STAT3 and YAP in liver cells and found that (i) STAT3 protein, expressed both in RLSCs and HepE14 cells, shows a nuclear localization only in progenitor cells; (ii) STAT3 and YAP are able to physically interact; (iii) STAT3 is recruited on Snail promoter in YAP-overexpressing hepatocytes (Supplementary Fig. S2). Starting from this data, we propose a new molecular cooperation of YAP and STAT3 in controlling gene expression of liver cells, which deserves further investigation.

### YAP is positively regulated by Snail at post-translational level

In our cell models, a reciprocal transcriptional control between HNF4α and Snail regulating EMT/MET dynamics and differentiation states was previously reported^24^. The experiments of YAP manipulation shown above demonstrated a positive and negative control of YAP on Snail and HNF4α transcription, respectively, suggesting that YAP could represent a further element of this epistatic circuitry. To further characterize this molecular interplay and the involvement of YAP in the circuitry, we firstly explored the capability of Snail to control, in turn, YAP expression. Literature data has shown functional cooperation between Snail and YAP^44,45^; however, nothing is known about their mutual control. After demonstrating a transcriptional regulation of YAP on Snail (data above) we have therefore set up experiments aimed at verifying the reciprocal control.

**Fig. 4:**
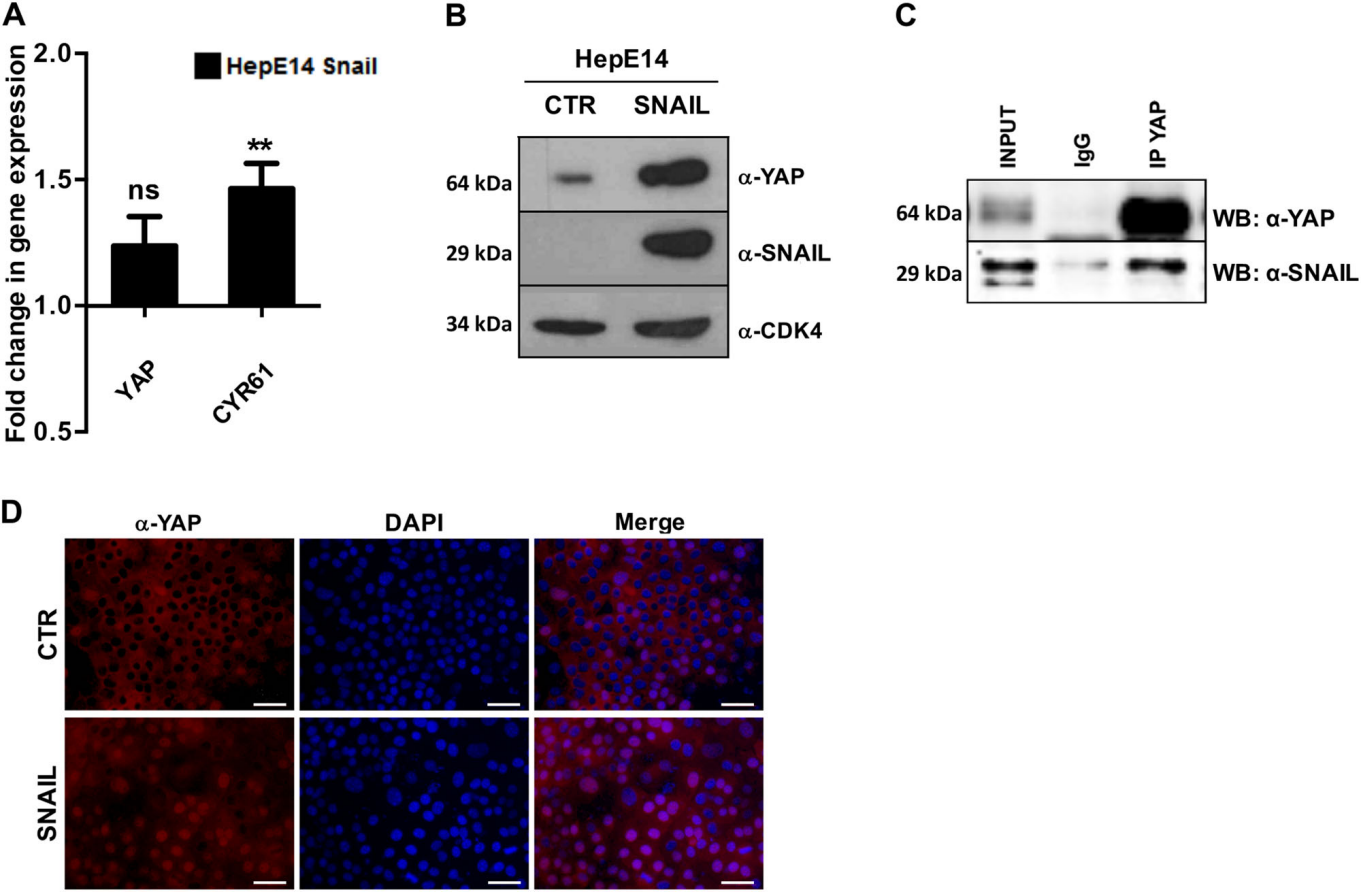
Snail positively controls YAP protein level and activity. **a** RT-qPCR analysis for YAP and its target gene CTGF in HepE14 infected with pLPCX/Snail (HepE14 Snail) or pLPCX (HepE14 CTR). The values are calculated by the 2^(−ΔCt)^ method, expressed as fold of expression versus the control (arbitrary value = 1) and shown as means ± S.E.M. of three independent experiments. Statistically significant differences are reported: **p < 0.01; ns = not significant). **b** Western blotting analysis for YAP and Snail in protein extracts from Snail-overexpressing HepE14 cells (SNAIL), compared with control cells (CTR). CDK4 was used as a loading control. **c** Co-immunoprecipitation of YAP and Snail in RLSCs. Cells were lysed, immunoprecipitated with anti-YAP antibody and then analyzed for Western Blotting with the indicated antibodies. As control, the immunoprecipitation with normal rabbit antiserum (IgG) was performed. **d** Immunofluorescence analysis of YAP (red) in HepE14 Snail, compared with HepE14 CTR. Nuclei were stained with DAPI (blue). Images are representative of three independent experiments. Scale bar: 50 µm

While the expression of an ectopic Snail in HepE14 cells induced only a slight up-modulation of YAP transcription (Fig. 4a), a massive increase of YAP protein, mainly located in the nucleus and active on its target gene Cyr61, has been observed (Fig. 4a, b, d). Notably, Snail and YAP physical interaction has been shown (Fig. 4c), that conceivably can play a role in the Snail-induced stabilization/nuclear translocation of YAP.

### YAP expression is steadily downregulated by HNF4α in hepatocytes

About HNF4α, while previous data reported its ability to interfere with YAP activity by competition with TEAD proteins on YAP target gene promoters^46^, a HNF4α-induced YAP gene regulation was not previously described.

**Fig. 5:**
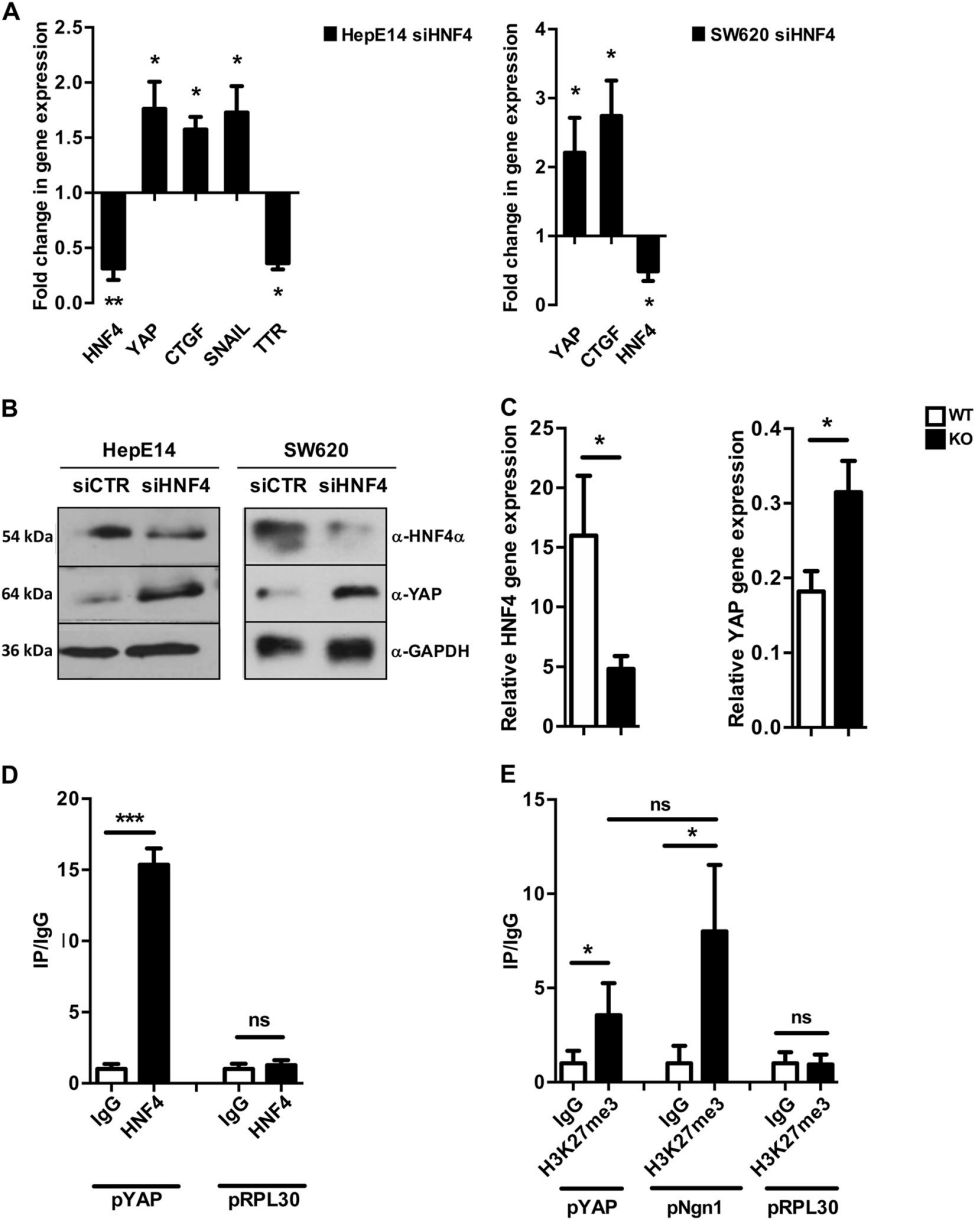
HNF4α negatively controls YAP expression and activity. **a** RT-qPCR analysis for the indicated genes in HNF4α-silenced HepE14 (HepE14 siHNF4) and colon SW620 cells (SW620 siHNF4), compared with GFP-silenced control cells. The values are calculated by the 2^(−ΔCt)^ method, expressed as fold of expression versus the control (arbitrary value = 1) and shown as means ± S.E.M. of at least three independent experiments. Statistically significant differences are reported (*p < 0.05; **p < 0.01). **b** Western blotting analysis for YAP and HNF4α in HNF4α-silenced HepE14 and SW620 cells (siHNF4), compared with GFP-silenced control cells (siCTR). GAPDH was used as a loading control. **c** RT-qPCR analysis of YAP and HNF4α in liver samples from four hepatocyte-specific HNF4α knockout (KO) mice^48^ and four wild-type (WT) mice. Statistically significant differences between the two groups of mice are reported (*p < 0.05). **d** qPCR analysis of ChIP assays with α-HNF4α antibody (HNF4), and as control, with normal rabbit IgG on chromatin from HepE14 cells. Values derived from at least four independent experiments are calculated as IP/IgG and reported as means ± S.E.M. respect to the IgG sample (arbitrary value = 1). RPL30 promoter was used as negative control (***p < 0.001; ns = not significant). **e** qPCR analysis of ChIP assays with α-H3K27me3 antibody (H3K27me3), and as control, with normal rabbit IgG on the chromatin from HepE14 cells. Values derived from at least three independent experiments are calculated as IP/IgG and reported as means ± S.E.M. respect to the IgG sample (arbitrary value = 1). RPL30 and Neurogenin promoters were used as negative and positive control, respectively (*p < 0.05; ns = not significant)

To analyze the possible role of HNF4α on the regulation of YAP gene expression, we performed experiments of HNF4α silencing in HepE14 cells that demonstrated an inverse correlation between HNF4α and YAP at transcriptional and protein level (Fig. 5a, b, left panels). Similar results have been obtained also in another cell type, SW620 colon cell line, in which we recently demonstrated a role of HNF4α in EMT/MET dynamics^47^ (Fig. 5a, b, right panels).

In extending our observations to an in vivo model, we exploited liver samples from hepatocyte-specific HNF4α knockout (KO) mice^48^. In this model, the loss of HNF4α has been obtained in adult healthy mice through use of the tamoxifen-inducible ErT2cre coupled to the serum albumin gene promoter. Also in this case, the loss of HNF4α correlated with a significant upregulation of YAP transcription (Fig. 5c). In this frame, it is worth recalling that livers from the acute HNF4α knockout mice were previously shown to exhibit hepatocyte dedifferentiation together with a marked induction of mesenchymal markers, highlighting the requirement of HNF4α in maintaining the hepatocyte identity by means a stable repression of the mesenchymal program^23^.

To investigate the direct involvement of HNF4α in the control of YAP transcription we analyzed the occupancy of YAP promoter. The in silico analysis of YAP promoter by Genomatix MatInspector revealed a putative HNF4α binding site located at −290 from TSS. ChIP assay performed in HepE14 hepatocytes revealed a significant recruitment of HNF4α to the corresponding chromatin fragment (Fig. 5d), and coherently with transcriptional results, a significant enrichment of the repressive histone modification H3K27me3 on HNF4α binding site of YAP promoter. Notably, the level of H3K27me3 on YAP promoter is comparable to that of the Neurogenin 1 gene promoter, here used as positive control (Fig. 5e).

Overall, our findings point to YAP as a new negative target gene of HNF4α, enforcing the notion that the stable repression of the mesenchymal program is required for the maintenance of epithelial phenotype of liver cells.

## Discussion

In this work, we demonstrated the functional role of YAP in the negative control of hepatocyte differentiation and unveiled the molecular mechanisms involved. We showed that the dynamic modulation of YAP expression (overexpression vs silencing) impacts on the induction/maintenance of the epithelial/hepatocyte identity controlling the expression of the MET/hepatocyte master gene HNF4α and of the EMT/stemness master gene Snail.

The functional interactions between YAP and HNF4α have been previously reported. Hippo signaling influences the redistribution of liver-specific transcriptional factors (including HNF4α and FoxA1) on a wide range of regulatory sequences^16^, affecting liver cell differentiation fate; moreover, a direct binding of YAP to an intronic regulatory sequence of HNF4α gene can be observed in liver cells from LATS1/2 knockout mice^41^. On the other side, HNF4α is able to negatively control YAP activity in liver cancer cells by competing with YAP for TEAD4 binding^46^. In accord with and in addition to these observations, we demonstrated that the functional link between YAP and HNF4α is also based on a reciprocal transcriptional inhibition through the direct occupancy of their own promoters.

Of note, we identified a new binding site for YAP/TEAD in the HNF4α promoter P1, different from the previously characterized site included in the first intron of the gene^41^. Furthermore, we showed for the first time a HNF4α steady-state binding and an inhibitory activity on YAP promoter in hepatocytes. We demonstrated that in hepatocytes HNF4α directly downregulates the expression of YAP gene, thus contributing to the repression of YAP-dependent mesenchymal program. Moreover, the observation of YAP upregulation in the in vivo model of HNF4α knockout mice strengthened the biological importance of the data obtained in cell lines. These findings reinforce the evidence that HNF4α, other than to activate and to maintain the epithelial/hepatocyte-specific program, actively and stably represses the mesenchymal one, downregulating Snail^23^ as well as YAP gene transcription to allow the fully expression of the differentiated phenotype.

A novelty of our work is also the identification of a reciprocal regulation between YAP and SNAIL. Previous reports suggested a functional cooperation between these proteins. Snail/Slug-YAP/TAZ complexes have been described in mesenchymal stem cells and during bone formation^44,45^. Furthermore, YAP was shown to cooperate with EMT master factor ZEB1 in the activation of the ZEB1-dependent cancer-promoting gene expression^49^. Moreover, an upregulation of Snail and YAP during TGFβ-induced EMT was previously described^31,50–52^. Here, we identified Snail as new transcriptional target gene of YAP. Indeed, by ChIP assays, we demonstrated Snail promoter occupancy by YAP, associated with a significant increase of the activating chromatin modification, the H3 histone acetylation. Our data is in accord with a previous report showing the transcriptional regulation by YAP of another member of Snail family, Snai2/Slug^53^.

In silico study of the Snail promoter region recruiting YAP did not reveal a consensus for TEAD while unveiled a STAT3 putative binding site, suggesting the involvement of this transcription factor in mediating the binding of YAP to DNA. Data from ChIP assay performed in YAP-overexpressing hepatocytes confirmed the recruitment of both STAT3 and YAP on Snail promoter. Further studies will be needed to define the role of STAT3 in the YAP-dependent transcriptional regulation in hepatocytes.

Importantly, we herein also showed that Snail in turn upregulates YAP at protein level and induces its nuclear localization, through a mechanism in which the physical interaction between the two proteins could play a role.

**Fig. 6:**
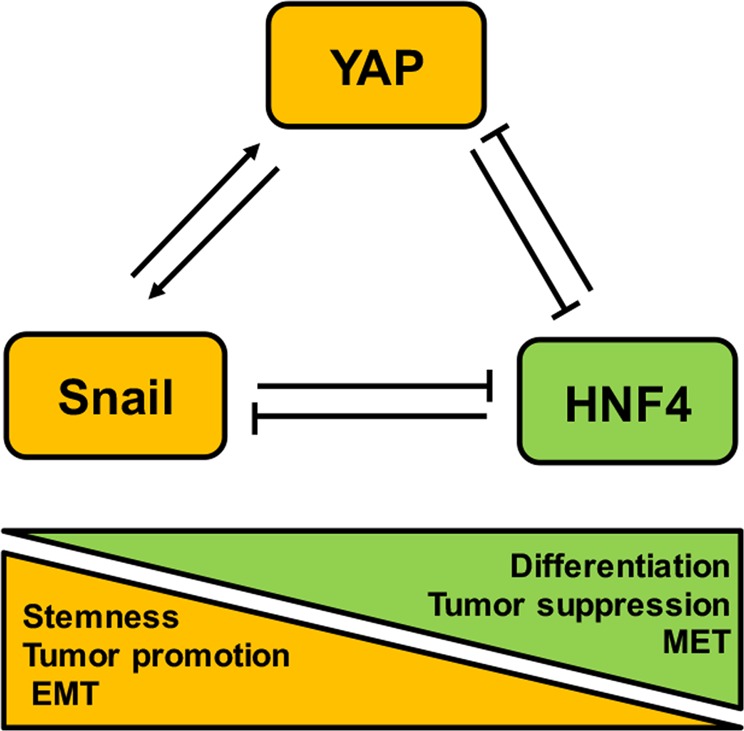
Suggested molecular circuitry controlling stemness/differentiation in liver cells, based on the reciprocal regulation among YAP, Snail, and HNF4α proteins. YAP protein has a direct role of in the repression of hepatocyte differentiation through the transcriptional upregulation of Snail and downregulation of HNF4α. Snail and YAP cooperate in the active repression of epithelial/hepatocyte differentiation through their reciprocal upregulation and the HNF4α downregulation. HNF4α actively and stably represses the mesenchymal program, downregulating both Snail and YAP. The same molecular interplay could be involved in different physiological and pathological cell outcomes

Overall, being HNF4α and Snail already described as components of an epistatic mini-circuitry of reciprocal repression, our results suggest that YAP could integrate this circuit influencing different liver cell outcomes (Fig. 6). This regulatory loop could be involved not only in the physiological maintenance/induction of differentiation states in liver cells but also, when dysregulated, in pathological cellular processes.

In conclusion, the finding of this work demonstrated that YAP is a new member of a molecular circuitry of reciprocal control between master factors, responsible for liver cell differentiation process. The demonstration that YAP is able to integrate itself into a tissue-specific molecular network shed new light on how transducers of several general stimuli can control tissue-specific cell outcomes.

## Materials and methods

### Cell lines and culture conditions

Resident liver stem cells (RLSCs) and hepatocytes E14 (HepE14) are immortalized and non-tumorigenic cell lines derived from murine liver explants at 14th days of development.

RLSCs are stem/precursor cells, displaying a typical stemness gene expression profile, self-renewing capability and multi-lineage differentiation potential both in culture and in vivo^35,36^. RLSCs were maintained at 37 °C, in a humidified atmosphere with 5% CO_2_ on collagen I (Collagen I, Rat Tail; Gibco-Life Technologies) coated dishes in Dulbecco’s modified Eagle’s medium (DMEM; Gibco-Life Technologies), supplemented with 10% fetal bovine serum (FBS), 2 mM glutamine (Gibco-Life Technologies) and antibiotics.

HepE14 are hepatocytes displaying a differentiated phenotype and a coherent gene expression profile. They have been used in a variety of studies of hepatocyte physiology being able to express a wide range of liver functions and products^34,54–58^. HepE14 cells were grown at 37 °C, in a humidified atmosphere with 5% CO2 on collagen I (Collagen I, Rat Tail; Gibco-Life Technologies) coated dishes in RPMI-1640 medium (Gibco-Life Technologies), supplemented with 10% FBS, 2 mM glutamine (Gibco-Life Technologies), 50 ng/ml EGF, 30 ng/ml IGF II (PeproTech), 10 µg/ml insulin (Roche) and antibiotics.

SW620 human colon-carcinoma cell line was grown in DMEM supplemented with 10% FBS (GIBCO^®^ Life Technology, Monza, Italy) and antibiotics.

### Cell transfections and retroviral infections

YAP-overexpressing HepE14 cells were obtained by transient transfection with pQCXIH-Myc-YAP5SA (gift from Kunliang Guan, Addgene plasmid # 33093)^7^ or the empty vector, by Lipofectamine 2000 transfection reagent (Invitrogen, Thermo Fisher) according to the manufacturer’s protocol. Cells were collected 48 h after transfection. Notably, YAP5SA protein, carrying mutations of LATS1/2-dependent phosphorylation sites (S61A, S109A, S127A, S164A, S381A), results constitutively active^7^.

Murine Snail and human HNF4α overexpressing cells were obtained by retroviral infection as previously described^21^. Snail recombinant retroviruses were produced in BOSC 23 packaging cells according to standard procedures by transient transfection of the retroviral construct pLPCX/Snail or pLPCX^21^. All viral particles were collected 48 h after transfection.

### RNA interference

Cells were transfected with equal amounts (100 pmol) of ON-TARGET plus SMARTpool Mouse YAP1 siRNA (22601; GE Healthcare Dharmacon, Lafayette, CO, USA), ON-TARGET plus SMARTpool Human HNF4α siRNA (GE Healthcare Dharmacon, Lafayette, CO, USA) or siRNA against GFP (5′-GGUGGUGACGAUCUGGGCUUUTT-3′) by Lipofectamine RNAiMAX (Invitrogen San Diego, CA) according to the manufacturer’s protocol. RNA and proteins were harvested and analyzed after 48 h.

### RT-qPCR

Total RNAs were extracted with Total RNA Mini Kit (Geneaid) according to manufacturer’s protocol and reverse-transcribed using PrimeScripte RT Master Mix (Perfect Real Time, Takara). cDNAs were amplified by qPCR reaction with GoTaq qPCR Master Mix (Promega) in Bio-Rad-iQ-iCycler. Relative amounts, calculated with the 2^(−ΔCt)^ method, were normalized with respect to the housekeeping gene RPL34 (60S ribosomal protein L34). The murine and human primers utilized are listed in Supplementary Tables 1 and 2, respectively.

### SDS-PAGE and Western Blotting

Cells were lysed in RIPA buffer containing freshly added cocktail protease inhibitors. Protein concentration was determined with Protein Assay Dye Reagent (Bio-Rad). Equal amounts of proteins were loaded on 12% acrylamide gels and then transferred to a nitrocellulose membrane (Bio-Rad). Blots were probed with the following primary antibodies: mouse monoclonal α-Snail (L70G2, Cell Signaling; 1:1000); mouse monoclonal α-YAP (SC-101199, Santa Cruz Biotechnology, inc.; 1:1000); goat monoclonal α-HNF4α (SC-6556 Santa Cruz Biotechnology, inc.; 1:1000); mouse monoclonal α-STAT3 (124H6, Cell Signaling; 1:1000); rabbit monoclonal α-CDK4 (C22, SC-260, Santa Cruz Biotechnology, inc.; 1:1000); mouse monoclonal α-GAPDH (MAB374, Millipore, Merck; 1:1000). Blots were then incubated with HRP-conjugated species-specific secondary antibodies (α-mouse IgG (H + L)-HRP Conjugated or α-Rabbit IgG (H + L)-HRP Conjugated from Bio-Rad; α-goat IgG (H + L) (705-036-147)-HRP Conjugated from Jackson immune Reasearch Laboratories, USA), followed by enhanced chemiluminescence reaction (WESTAR Nova 2.0 or WESTAR etaC, Cyanagen).

### Immunofluorescence analysis

For indirect immunofluorescence analysis, cells were fixed in 4% paraformaldehyde, permeabilized with 0.2% Triton-X100 and incubated with the following primary antibodies: mouse monoclonal α-YAP (SC-101199, Santa Cruz Biotechnology, inc.; 1:50); mouse monoclonal α-Snail (L70G2, Cell Signaling Technology; 1:50); rabbit monoclonal α-Vimentin (ab92547, Abcam, Cambridge, UK; 1:400); mouse monoclonal α-E-cadherin (BD 610182, BD Biosciences Pharmingen, Palo Alto, CA, USA; 1:50); mouse monoclonal α-STAT3 (124H6, Cell Signaling; 1:50). Secondary antibodies: α-mouse Alexa-Fluor 594 and α-rabbit Alexa-Fluor 488 (Molecular Probes, Eugene, OR, USA; 1:400). Nuclei were stained with DAPI (Calbiochem Merck, Darmstadt, Germany). Preparations were examined under Nikon Eclipse fluorescent microscope equipped with a CCD camera (Nikon Inc.). Digital images were acquired by Nikon NIS elements software (Nikon Corporation) and processed with Adobe Photoshop 7 software (Adobe Systems, Mountain View, CA). The same enhanced color levels were applied for all channels.

### Chromatin Immunoprecipitation (ChIP)

ChIP assays were performed as previously reported^21^ by using 5 µg rabbit α-YAP (H-125X, Santa Cruz Biotechnology Inc.), rabbit α-HNF4Α (H-171X, Santa Cruz Biotechnology), mouse α-STAT3 (124H6, Cell Signaling) or the negative control rabbit IgG (Millipore Corp., Bedford, MA, USA). Equal amounts of immunoprecipitated DNA and relative controls were used for qPCR analysis with the following primers: *HNF4α* promoter, forward 5′-CGGTTCCCAAAGCATGTGAC-3′ and reverse 5′-ATAAAGCTGTCCTGGGTCGC-3′; *Snail* promoter, 5′-TGTTCAGGGCTGTGTAGAC-3′ and reverse 5′-GAGCTGCTGACCTTTGG-3′; *YAP* promoter, forward 5′-ACCTTAGTGCGGGTGAACAG-3′ and reverse 5′-GTCGCTACATTCCTGCAGAC-3′, *CTGF* promoter, forward 5′-CAATCCGGTGTGAGTTGATG-3′ and reverse 5′-GGCGCTGGCTTTTATACG-3′, *RPL30* promoter, forward 5′-TAAGGCAGGAAGATGGTGG-3′ and reverse 5′-CAGTGTGCTCAAATCTATCC-3′; Neurogenin 1, forward 5′-CCTCCCGCGAGCATAAATTA-3′ and reverse 5′- GCGATCAGATCAGCTCCTGT-3′. qPCR analysis of immunoprecipitated samples (IP) and of negative control (IgG) were normalized to total chromatin input and expressed as (IP/IgG)/Input.

For the analysis of histone modifications (Histone ChIP), chromatins were immunoprecipitated with 5 μg of Anti-trimethyl-Histone H3 (Lys27) (07-449; Millipore Corp., Bedford, MA, USA), or Anti-acetyl-Histone H3 Antibody (06-599; Millipore Corp., Bedford, MA, USA) or rabbit IgG (Millipore Corp., Bedford, MA, USA) by using Magna ChIP protein A magnetic beads (Millipore). The immunoprecipitated DNA was amplified by qPCR. Data were expressed as (IP/IgG)/Input and normalized respect to the immunoprecipitation efficiency, evaluated through the qPCR of the promoter of the housekeeping gene RPL30 (acetylation) or the promoter of Neurogenin 1, a gene not expressed in the liver (trimethylation).

### Statistical analysis

Paired one-tailed *t*-test and Excel function were used for statistical analyses of at least three independent experimental replicates. The Mann–Whitney *U* test was used to compare differences between the two groups of mice (knockout vs wild type). *P*-values (*p*) < 0.05 were considered statistically significant (*p < 0.05; **p < 0.01; ***p < 0.001).

## Conflict of interest

The authors declare that they have no conflict of interest.

## Supplementary information

Supplementary Information

Supplementary Figure S1

Supplementary Figure S2
