# Supplementary material for: YAP integrates the regulatory Snail/HNF4α circuitry controlling epithelial/hepatocyte differentiation
Source: Cell Death Dis. 2019 Oct 10;10(10):768. doi: 10.1038/s41419-019-2000-8 (PMC6787001; doi:10.1038/s41419-019-2000-8)
Supplement: Supplementary file 1 — Supplementary Information [file 41419_2019_2000_MOESM1_ESM.docx]

**Supplementary Information**

**YAP integrates the regulatory Snail/HNF4α circuitry controlling epithelial/hepatocyte differentiation**

Valeria Noce, Cecilia Battistelli, Angela Maria Cozzolino, Veronica Consalvi, Carla Cicchini, Raffaele Strippoli, Marco Tripodi, Alessandra Marchetti and Laura Amicone

**Supplementary Figure Legends**

**Supplementary Fig. 1** YAP overexpression in HepD3 (Amicone et al., 1997) and AML12 (ATCC, CRL-2254, American Tissue Culture Collection) murine hepatocytes. RT-qPCR analysis for the indicated genes in cells transiently transfected with pQCXIH-Myc-YAP-5SA (YAP5SA), compared with control cells transfected with empty vector. The values are calculated by the 2(−ΔCt) method, expressed as fold of expression versus the control (arbitrary value=1) and shown as means ± S.E.M. of three independent experiments. Statistically significant differences are reported (*p<0.05).

**Supplementary Fig. 2** Analysis of STAT3 expression/activity. (A) Immunofluorescence analysis for STAT3 (red) in RLSC and HepE14 cell lines. Nuclei were stained with DAPI (blue). Scale bar: 50µm. (B) Western blot analysis for YAP and STAT3 in RLSC and HepE14 cell lines. GAPDH was used as loading control. (C) Co-immunoprecipitation of YAP and STAT3 in RLSCs. Cells were lysed, immunoprecipitated with anti-YAP antibody and then analyzed for Western Blotting with the indicated antibodies. As control, the immunoprecipitation with normal rabbit antiserum (IgG) was performed. TCE= total cell extracts. (D) qPCR analysis of ChIP assays with anti-STAT3 antibody and, as control, with normal rabbit IgG on chromatin from HepE14 CTR or HepE14 YAP5SA. Values derived from three independent experiments are calculated as IP/IgG and reported as means ± S.E.M. respect to the control sample (arbitrary value=1). RPL30 promoter was used as negative control. (*p<0.05).

**Supplementary Tables**

**Supplementary Table 1. List of mouse primers used for RT-qPCR experiments.**

| **Gene** | **Forward primer (5’-3’)** | **Reverse primer (5’-3’)** |
| --- | --- | --- |
| YAP | CTGGACCCTCGTTTTGCCAT | TATTTGCTGCTGCTGGTTGG |
| Cyr61 | AGAGGCTTCCTGTCTTTGGC | CCAAGACGTGGTCTGAACGA |
| CTGF | ATCATGCTCGCCTCCGTCGC | TAGCAGGCCGGGTGCAGAGA |
| DDIT4 | GCCGGAGGAAGACTCCTCATA | CATCAGGTTGGCACACAGGT |
| HNF4α | TCTTCTTTGATCCAGATGCC | GGTCGTTGATGTAATCCTCC |
| Albumin | TTCCTGGGCACGTTCTTGTA | GCAGCACTTTTCCAGAGTGG |
| ApoC3 | GGACGCTCCTCACTGTGG | CACGACTCAATAGCTGGAG |
| Ecad | CTACTGTTTCTACGGAGGAG | CTCAAATCAAAGTCCTGGTC |
| TTR | CCATGAATTCGCGGATGTGG | TCAATTCTGGGGGTTGCTGA |
| Occludin | ACCTGATGAATTCAAACCCA | GTGAAGAATTTCATCTTCCGG |
| Snail | CCACTGCAACCGTGCTTTT | CACATCCGAGTGGGTTTGG |
| RPL34 | GGAGCCCCATCCAGACTC | CGCTGGATATGGCTTTCCTA |

**Supplementary Table 2. List of human primers used for RT-qPCR experiments.**

| **Gene** | **Forward primer (5’-3’)** | **Reverse primer (5’-3’)** |
| --- | --- | --- |
| CTGF | AGGAGTGGGTGTGTGACGA | CCAGGCAGTTGGCTCTAATC |
| HNF4α | CATGGACATGGCCGACTACA | ATTGCCCATCGTCAACACCT |
| YAP | AGGTTGGGAGATGGCAAAGA | ACCTGAAGCCGAGTTCATCA |
| RPL32 | GGAGCGACTGCTACGGAAG | GATACTGTCCAAAAGGCTGGAA |
