# Supplementary figures and images for: YAP integrates the regulatory Snail/HNF4α circuitry controlling epithelial/hepatocyte differentiation
Source: Cell Death Dis. 2019 Oct 10;10(10):768. doi: 10.1038/s41419-019-2000-8 (PMC6787001; doi:10.1038/s41419-019-2000-8)

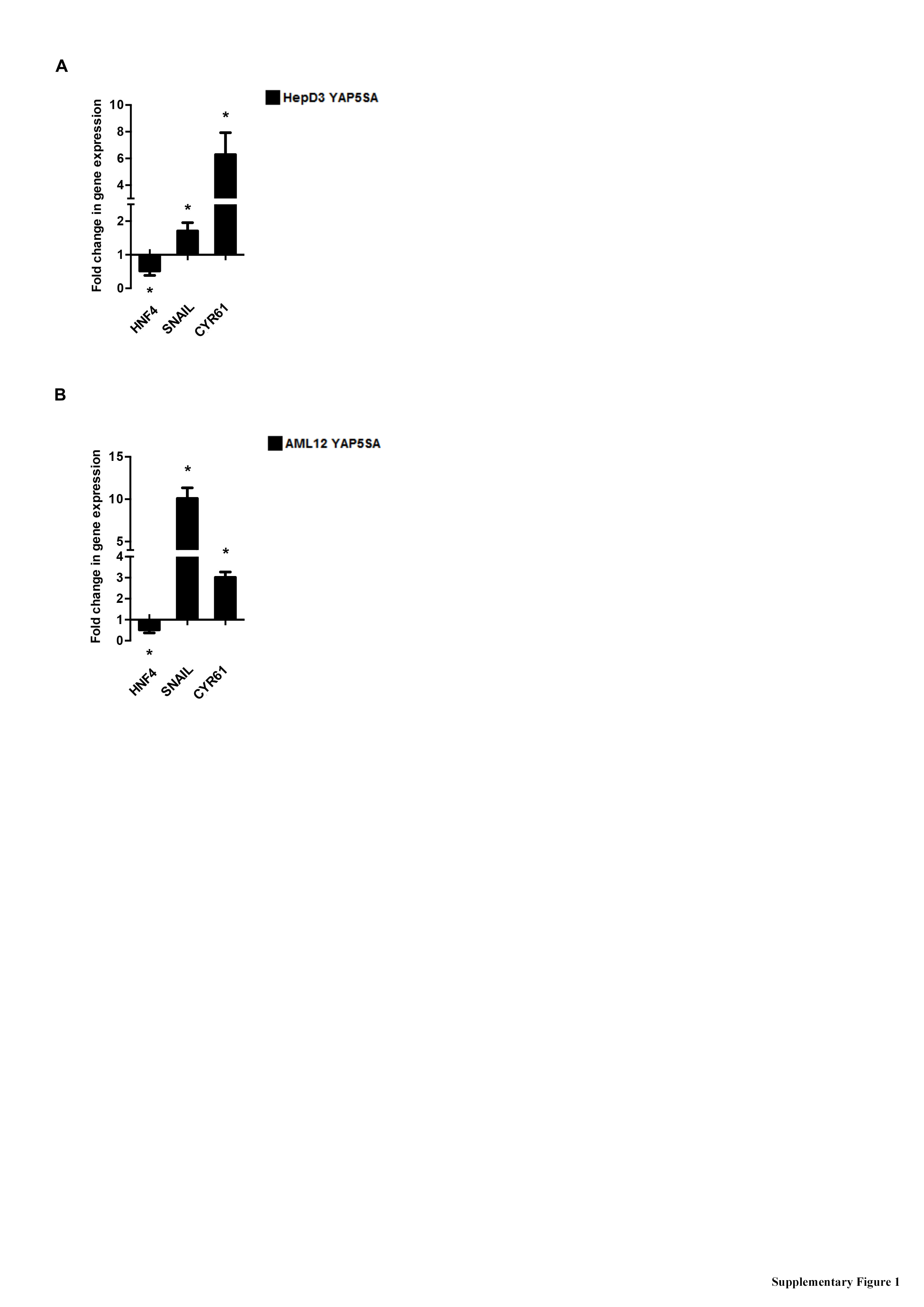

Supplement: Supplementary file 2 — Supplementary Figure S1 [file 41419_2019_2000_MOESM2_ESM.tif]

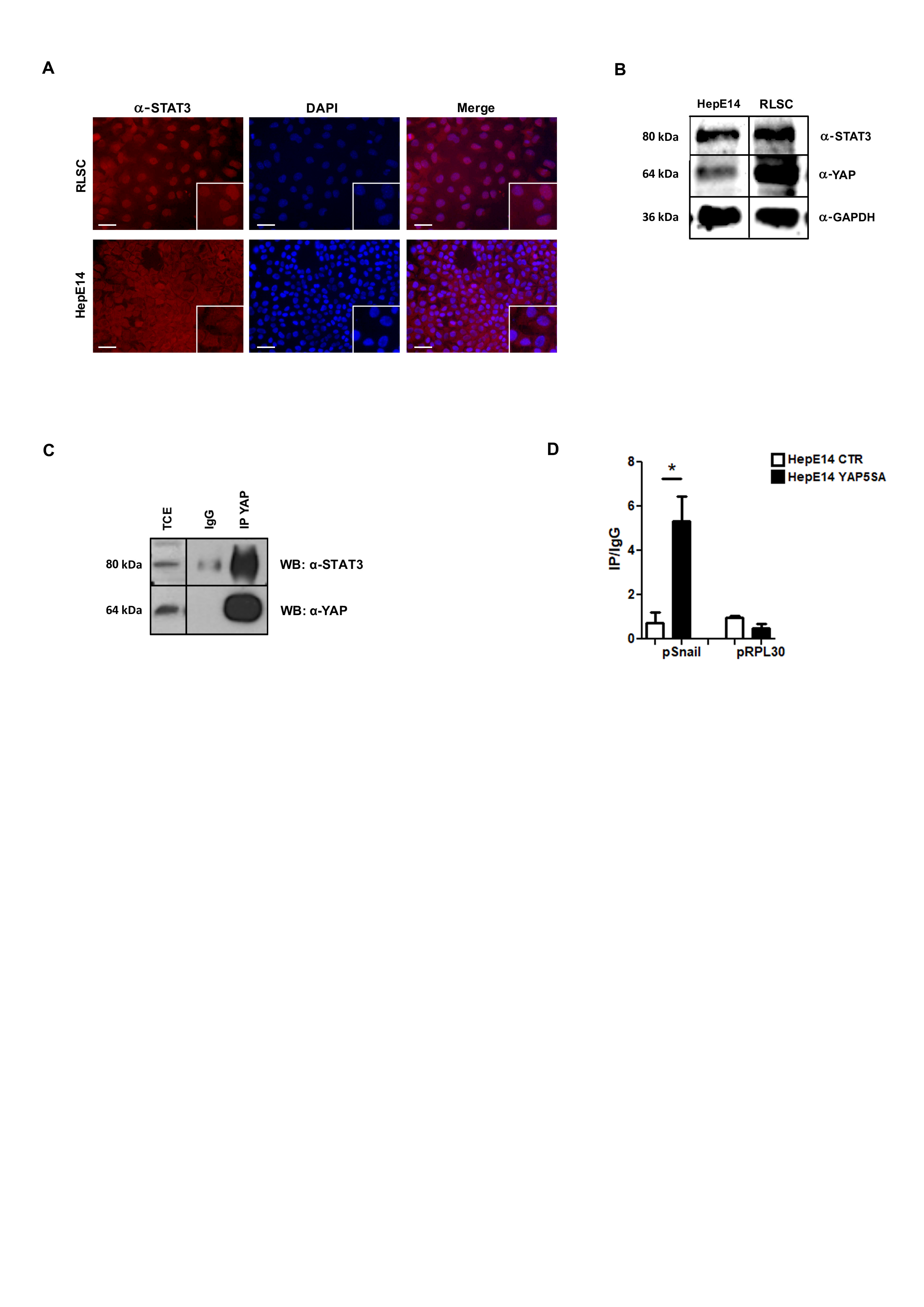

Supplement: Supplementary file 3 — Supplementary Figure S2 [file 41419_2019_2000_MOESM3_ESM.tif]
